# Antifibrotic Effects of Kangxian Ruangan Capsule on Rats with Nonalcoholic Fatty Liver Fibrosis and Hepatic Stellate Cells through Regulation of TGF-*β* and TLR4 Signaling Pathways

**DOI:** 10.1155/2021/5649575

**Published:** 2021-08-10

**Authors:** Liming Liu, Ying Zhou, Dan Dai, Hongmei Xia, Kang Zhao, Jianjun Zhang

**Affiliations:** Department of Liver Medicine, Hubei No. 3 People's Hospital, Jianghan University, Wuhan, Hubei, China

## Abstract

Kangxian ruangan (KXRG) is a traditional Chinese medicine (TCM) formula consisting of 12 herbs. TCM syndrome differentiation proposes that KXRG exerts pharmacological effects against nonalcoholic fatty liver disease (NAFLD) fibrosis. This work investigates the effect of KXRG on NAFLD fibrosis *in vivo* and *in vitro*. *In vivo*, the NAFLD fibrosis model was constructed in Wistar rats using methionine- and choline-deficient (MCD) diet, followed by KXRG (0.92 g/kg/d) treatment for 8 weeks. *In vitro*, primary hepatic stellate cells (HSCs) were activated using platelet-derived growth factor (PDGF) and treated with KXRG. Molecular mechanisms underlying fibrosis were investigated. After 8 weeks, compared with the control groups, the histological lesions, degree of fibrosis, and inflammatory reaction increased with the MCD diet as demonstrated by histological changes and increased fibrosis-related (*α*-SMA, TGF-*β*, COL1A1, and desmin, *P* < 0.01) and inflammation-related factors (TNF-*α*, MCP-1, and F4/80, *P* < 0.01), whereas they decreased with KXRG treatment (*P* < 0.01). KXRG not only inhibited the proliferation of activated HSCs and promoted their apoptosis but also resulted in G0-G1 arrest. Furthermore, KXRG suppressed HSC activation (*P* < 0.01), collagen synthesis (*P* < 0.01), and *α*-SMA expression (*P* < 0.01) with PDGF stimulation. In both the MCD diet-induced animal model and PDGF-induced cell model, KXRG inhibited TGF-*β* and TLR4 signaling (*P* < 0.01), similar to corresponding small-molecule inhibitors. These results demonstrated that KXRG might exert suppressive effects against NAFLD fibrosis via regulating TGF-*β* and TLR4 signaling. KXRG may act as a natural and potent therapeutic agent against NAFLD.

## 1. Introduction

Nonalcoholic fatty liver disease (NAFLD) is a spectrum of liver pathology that is strongly associated with obesity, diabetes, and cardiovascular disease [1]. Described as the typical hepatic manifestation of metabolic syndrome, NAFLD is now the most common cause of chronic liver diseases worldwide [2]. Epidemiologic findings indicate that the prevalence of NAFLD varies depending on population, ethnicity, and social formation, but increasing data suggest that its prevalence in the adult population in many Asian countries may be as high as that in the West [3, 4]. The majority of people affected by NAFLD only experience simple steatosis or slight inflammatory response, which confer relatively low risk [5]. However, without prompt and proper treatment, the vast majority of patients with progressive NAFLD will develop complications, including steatohepatitis [6], fibrosis [7], cirrhosis [8], end-stage liver failure [9], and malignant hepatocellular carcinoma [10]. In this case, patient health will be significantly threatened.

At present, the pathogenesis of NAFLD has not yet been determined, but a multiple-strike theory has revealed that NAFLD is caused by the combination of diet, genetics, and intestinal microenvironment [11]. Meanwhile, the degree of fibrosis is a critical feature that indicates the prognosis of NAFLD patients. Fibrosis is not only a common sign of the development of chronic liver diseases [12] but also the only way to further develop liver cirrhosis [13] and even liver cancer [14]. Recent clinical data have placed increasing importance on identifying fibrosis, as it is a strong indicator of hepatic disease-related mortality [15]. Therefore, the effective reduction of fibrosis is the key to treat NAFLD.

Hepatic stellate cells (HSCs) are acknowledged as the cytological basis of liver fibrosis characterized by excess deposition of the extracellular matrix (ECM), and the fibrogenic process involves multiple cellular and molecular pathogenetic mechanisms [7, 16]. As a central event in the development of hepatic fibrosis, HSCs are activated by liver injury, usually with changes in gene expression and major phenotypical transformation to *α*-smooth muscle actin (*α*-SMA)-positive myofibroblasts that increase malignant biological characteristics [17], produce large amounts of collagen and other ECM molecules [18], and secrete proinflammatory cytokines such as tumor necrosis factor- (TNF-) *α* and interleukin- (IL-) 1*β* [19]. Numerous studies have shown that the stimulation of several intracellular signaling cascades is associated with HSC activation. For example, transforming growth factor- (TGF-) *β*1 activates Smad 2/3 phosphorylation and the transcript levels of plasminogen activator inhibitor-1 and matrix metalloproteinase-2 as well as autophagy in HSCs, showing the important regulatory effect of the TGF-*β*/Smad signaling pathway on HSC activity [20]. In addition, platelet-derived growth factor (PDGF), a potent fibrogenic cytokine, activates the mitogen-activated protein kinase/extracellular signal-regulated kinase and phosphatidylinositol 3-kinase/AKT/70-kDa ribosomal S6 kinase signaling pathways, further regulating HSC proliferation and migration [21, 22]. Hence, suppressing HSC activity and ensuring hepatocyte activity via regulating various intracellular signaling cascades are vital means of treating liver fibrosis.

As a generally accepted form of complementary and alternative medicine, traditional Chinese medicine (TCM) has been widely reported to be useful and has few side effects in many diseases including liver fibrosis [23]. Hu et al. showed that the TCM forsythiae fructose suppresses liver fibrosis by regulating TGF-*β*/Smads and TLR4/MyD88/NF-*κ*B signaling pathways [24]. Cai et al. found that the TCM Yinchenhao decoction attenuates liver fibrosis through modulating TGF-*β*/Smad/ERK signaling and bile acid metabolism [25]. After thousands of years of practice, many herbal drugs and formulae in TCM, particularly classical TCM formulae, are still used in modern Chinese medicine. TCM adopts a holistic view of disease prevention and treatment with “treatment with syndrome differentiation” as the basic underlying principle, implying that the composition of formula is based on the symptoms and signs of patients analyzed. Thus, TCM formulae are the main form of therapy in TCM. For the pathogenesis of NAFLD, TCM believes that deficiency of the spleen, damp heat, and phlegm stasis are the key factors. Based on the pharmacological principles of TCM, Kangxian ruangan (KXRG) has the functions of replenishing the spleen and warming the yang. Thus, judging from the syndrome differentiation of TCM, it is reasonable to hypothesize the modern pharmacological effect of KXRG capsules in terms of “replenishing the spleen and warming the yang.”

In this study, a rat model of NAFLD fibrosis was established by inducing a methionine- and choline-deficient (MCD) diet. The therapeutic effect of KXRG capsules with an optimum concentration on model rats was evaluated through preliminary experiments. Therewith, primary HSCs were isolated and cultured *in vitro*, and we investigated the effects of KXRG capsules on PDGF-mediated HSC activation. In addition, we sought to confirm that KXRG capsules reduce HSC activation by suppressing the TGF-*β* and toll-like receptor (TLR) 4 signaling pathways with the aim of providing more targeted natural medicine for the treatment of NAFLD fibrosis and severe liver diseases.

## 2. Materials and Methods

### 2.1. Preparation of Medication

Kangxian ruangan capsule, a well-known traditional Chinese herbal medicine, is a classical formula containing king drug: *Artemisia capillaris* (20 g), *Astragalus membranaceus* (10 g), *Turtle shell* (10 g); minister drug: *Coix seed* (20 g), *Salvia miltiorrhiza* (30 g), *Angelica sinensis* (10 g), *Curcuma zedoaria* (10 g), *Ground beetle* (10 g); assistant drug: *Panax notoginseng* (6 g), peach seed (10 g), *Parched pangolin scales* (6 g), ambassador drug: *Rhizoma atractylodis macrocephalae* (10 g). All herbs were purchased from Hubei Tianji Chinese Herbal Sliced Medicine Co., Ltd. (Wuhan, China), and the Department of Pharmacy, the Third People's Hospital of Hubei Province, is responsible for drug safety control. Based on the traditional decoction preparation method, the decoction was concentrated and prepared as a capsule (the content of crude drugs: 0.046 g/mL) at the Pharmacy Department of the Hubei Provincial Hospital of TCM.

### 2.2. Rats and Treatments

Twenty-four specific pathogen-free male Wistar rats (age: 7–8 weeks; weight: 250–280 g) were purchased from the Animal Center of Hubei (No. 42000600013965, Hubei, China). All experimental procedures were approved by the Animal Ethics Committee of the Hubei Provincial Hospital of TCM. We followed the methods of [26]. All rats were maintained under constant temperature (22 °C) and humidity (50% ± 15%) in a 12-h light/dark cycle, given free access to deionized water and fed irradiated disinfectant food. After one week of adaptive feeding, the rats were randomly divided into 3 groups (8 rats in each group): (1) control group (CTRL); (2) model group (MOL); (3) Kangxian ruangan (KXRG) group. In this study, the rat model of NAFLD fibrosis was established by the methionine- and choline-deficient (MCD) diet [27–29]. The rats in the CTRL group were fed methionine and choline supplement (MCS) fodder, and those in the other groups were fed MCD fodder (Trophic Animal Feed High-tech Co., Ltd., Jiangsu, China). The rats in the treatment group were gavaged with the drug diluted in the normal saline for 8 weeks (KXRG group: 0.92 g/kg/d, the content of crude drugs: 0.046 g/mL). The drug treatment started with the NAFLD fibrosis induction. The rats in the CTRL and MOL groups were gavaged with the same volume (2 mL/100 g/d) of normal saline for 8 weeks.

### 2.3. Enzyme-Linked Immunosorbent Assays (ELISA)

8 weeks after treatment, all rats were anesthetized by 60 mg/kg pentobarbital (intraperitoneal injection, no. P3761, Sigma-Aldrich, USA) after 12 h of fasting and blood samples were collected from the abdominal aorta of the rats. The sera was separated and collected by ×1000 g centrifugation at 4°C. The levels of TNF-*α* and IL-6 in serum were determined by corresponding commercial ELISA kits according to the manufacturer's instructions (TNF-*α*: no. MU30030; IL-6: no. MU30044, Bioswamp, China). Absorbance was detected with a microplate reader (Bio-Rad, USA) at 450 nm, and the values of various indicators were calculated by a standard curve. In addition, the total cholesterol (TC) and triglyceride (TG) and the levels of alanine aminotransferase (ALT) and aspartate aminotransferase (AST) in sera were determined using a fully automatic biochemical analyzer.

### 2.4. Quantitative Reverse Transcription Polymerase Chain Reaction (qRT-PCR) Analysis

Total RNA of the liver tissues were extracted using TRIzol (Ambion, Texas, USA) and reversed-transcribed into cDNA. The collected cDNA was then subjected to amplification using a CFX Connect 96 (Bio-Rad, USA). The gene primers are as follows: TNF-*α*, forward, 5′'- CCACGCTCTTCTGTCTACTG-3′', reverse, 5′'- GCTACGGGCTTGTCACTC-3'; MCP-1, forward, 5′'- ACCCCAATAAGGAATG-3′', reverse, 5′'- GGTGGTTGTGGAAAAG-3'; F4/80, forward, 5′'- TTTCAGCTCTCGCAACA-3′', reverse, 5′'- GGTCAGCAACCTCGTATC-3'; TGF-*β*, forward, 5′'- AGGAGACGGAATACAGGG-3′', reverse, 5′'- ATGAGGAGCAGGAAGGG-3'; COL1A1, forward, 5′'- GTCCCAACCCCCAAAA-3′', reverse, 5′'- CCAGGCACGGAAACTC-3'; Desmin, forward, 5′'- AGGAAATCCAACTGAGAGA-3′', reverse, 5′'- CAATTCGAGCCAGAGTG-3'; GAPDH, forward, 5′'- CAAGTTCAACGGCACAG-3′', reverse, 5′'- CCAGTAGACTCCACGACAT-3'. GAPDH served as endogenous control. The 2^-△△Ct^ method was performed for the calculation of the relative miRNA and mRNA expression levels [30].

### 2.5. Histopathological Examination and Immunohistochemical Staining

After the rats were sacrificed, a portion of fresh liver tissues was removed and fixed with 4% paraformaldehyde for 24 h, embedded in paraffin, cut into sections of 5-6 *μ*m in thickness, and subjected to hematoxylin and eosin (H&E) staining to evaluate histological changes. Images of the stained slides were captured with a microscope (Olympus, Tokyo, Japan). The degree of fibrosis has been evaluated by a semiquantitative scoring system (SSS). The higher the score, the more severe the degree of fibrosis [31]. Immunohistochemical staining was carried out to detect the expression of *α*-SMA in the liver using rabbit polyclonal anti-*α*-SMA as the primary antibody (no. ab5694, 1 : 200, Abcam, Cambridge, UK) and horseradish peroxidase- (HRP-) conjugated goat anti-rat IgG (no. ab6721, 1 : 1000, Abcam) as the secondary antibody. All staining was visualized and photographed under an ordinary optics microscope after color reaction of the diaminobenzidine (DAB) kit (no. DA1010, Solarbio, Beijing, China). Subsequently, the proportion of *α*-SMA-positive regions was analyzed by ImageJ software (NIH Bethesda, MD, USA). The remaining fresh liver tissues were stored at −80°C for subsequent molecular assays.

### 2.6. HSC Culture, Identification, and Treatment

Primary HSCs were prepared from SPF male Wistar rats according to a previously described method [32]. Primary HSCs were cultured in Dulbecco's modified Eagle medium (DMEM) supplemented with 10% fetal bovine serum (FBS) for 14 days, and activated HSCs were identified by immunocytochemical staining for *α*-SMA and used in further experiments. To evaluate the effect of KXRG on HSC activation (MTT method), the cells were serum-starved overnight and cultured for 48 h in the presence (2.5, 5, and 10 mg/mL) or absence (0 mg/mL) of KXRG and 12, 24, and 48 h for cell proliferation analysis. The toxicity detection of KXRG on normal liver parenchymal L02 cells was also performed (MTT method). In a similar way, the effects of different concentrations of KXRG on cell apoptosis, cell cycle, and proliferation capability in cells stimulated by different concentrations of PDGF-BB (0, 1, 5, 10, and 20 ng/mL) were also examined. To further evaluate the effects of KXRG on proliferation ability and collagen synthesis capability in PDGF-induced HSCs, a model of HSC fibrosis induced by 10 ng/mL PDGF was constructed based on previous studies [33]. To determine the effect of KXRG on intracellular signaling, the model group was treated with two molecular inhibitors. For the inhibition of the TGF-*β* signaling pathway, the TGF-*β* receptor inhibitor LY2109761, a TGF- receptor type I/II dual inhibitor [34], was used. For the inhibition of the TLR4 signaling pathway, the TLR4 small-molecule inhibitor TAK-242 was applied. HSCs were serum-starved overnight and split into the following groups: control; model (10 ng/mL PDGF); KXRG treatment (10 ng/mL PDGF + 10 mg/mL KXRG); LY2109761 treatment (10 ng/mL PDGF + 10 *µ*M LY2109761); TAK-242 treatment (10 ng/mL PDGF + 1 *µ*M TAK-242). After 24 h of treatment, the cells were harvested for western blot.

### 2.7. Cell Proliferation Assay

Cell proliferation was determined using an MTT assay kit (No. C0009, Beyotime, Shanghai, China). Primary activated HSCs and L02 cells were cultured overnight. KXRG (0, 2.5, 5, and 10 mg/mL) was added to the medium and the cells were cultured for an additional 48 h. The absorbance was measured at 12, 24, and 48 h. Changes in the proliferation of PDGF-induced cells at different PDGF concentrations (0, 1, 5, 10, and 20 ng/mL) were evaluated under a similar operation. In addition, the effect of KXRG on PDGF-induced cell proliferation was elucidated.

### 2.8. HSC Apoptosis Assay

Primary activated HSCs were treated with KXRG (0, 2.5, 5, and 10 mg/mL) for 24 h and harvested for the apoptosis assay using the Annexin V-fluorescein (FITC)/propidium iodide (PI) double-labeled flow cytometry kit (no. CA1020, Solarbio, Beijing, China). Data were acquired by a FC500 MCL flow cytometer (Beckman, USA) to evaluate the apoptotic rate (*B*2 + B4 quadrant) of each sample.

### 2.9. HSC Cycle Assay

At 24 h after KXRG treatment (0, 2.5, 5, and 10 mg/mL), the cells were fixed in ice-cold 75% ethanol, washed, and labeled with PI from the PI staining cell cycle detection kit (No. KGA512, Keygen, Jiangsu, China). Flow cytometry was carried out with a FC500 MCL flow cytometer to analyze the changes in cell cycle progression.

### 2.10. Evaluation of Collagen Synthesis

HSCs were cultured overnight in DMEM supplemented with 10% FBS. PDGF (10 ng/mL) was then added with 10 mg/mL KXRG and the cells were cultured for an additional 48 h. Collagen synthesis was evaluated by measuring the amount of ^3^H-proline incorporation as previously described [35]. The results are expressed as Cpm per 10^5^ cells.

### 2.11. Immunocytochemical Staining

HSCs were cultured overnight in DMEM supplemented with 10% FBS. PDGF (10 ng/mL) was then added with 10 mg/mL KXRG and the cells were seeded in two-well chamber slides and cultured for an additional 24 h. Thereafter, the cells were fixed in 4% formaldehyde containing 2% sucrose for 10 min and permeabilized with phosphate-buffered saline containing 0.5% Triton X-100 (Sigma-Aldrich) for 5 min. The *α*-SMA primary antibody (Abcam) was diluted at 1 : 200, and the secondary antibody (HRP-conjugated goat anti-rat IgG) (Abcam) was diluted at 1 : 1000. Positive cells were counted after color reaction of DAB using an optical microscope (Olympus, Tokyo, Japan).

### 2.12. Western Blot Analysis

The total proteins of liver tissues and HSCs were split using a total protein extraction kit (no. W034, Jiancheng Bioengineering Institute, Nanjing, China) and the protein concentration was assessed using a bicinchoninic acid method. Approximately 30 *µ*g of total proteins were electrophoresed in 12% polyacrylamide gels and the separated proteins were transferred to polyvinylidene difluoride membranes. Nonspecific binding was blocked with 5% skimmed milk for 2 h at 37°C. The membranes were then incubated with monoclonal or polyclonal antibodies against TGF-*β*1 (no. ab92486, 1 : 500, Abcam), TGF-*β*2 (no. ab113670; 1 : 1000, Abcam), Smad2 (no. ab63576, 1 : 1000, Abcam), phosphorylated- (p-) Smad2 (no. ab53100, 1 : 500, Abcam), Smad3 (no. ab122028 1 : 500, Abcam), p-Smad3 (no. ab193297, 1 : 500, Abcam), Smad7 (no. PA5-46373, 1 : 500, Thermo, USA), TLR4 (no. 48–2300, 1 : 200, Thermo), MyD88 (no. ab2064, 1 : 1000, Abcam), and NF-*κ*B p65 (no. ab16502, 1 : 2000, Abcam) at 4°C overnight. GAPDH (no. ab9485, 1 : 1000, Abcam) was used as a control. Next, the membranes were incubated with HRP-conjugated goat anti-rabbit secondary antibody IgG (no. ab6721; 1 : 5000, Abcam) for 1 h at room temperature. Images were obtained using a multifunctional Gel Imaging System (Image Quant LAS 500, General Electric, Fairfield, CT, USA), and the gray value of each band was quantified and analyzed by IQTL 8.1 software (gamma ratio/GAPDH).

### 2.13. Statistical Analyses

All values are presented as the mean ± standard deviation. The *t*-test was used for comparison between two groups. One-way analysis of variance followed by Tukey's post hoc test was performed to compare between three or more groups using SPSS 19.0 software (IBM Corp., Armonk, NY, USA). *P* < 0.05 was considered as a statistically significant difference.

## 3. Results

### 3.1. The Effect of KXRG on Inflammatory Factors and Histopathology

**Figure 1:**
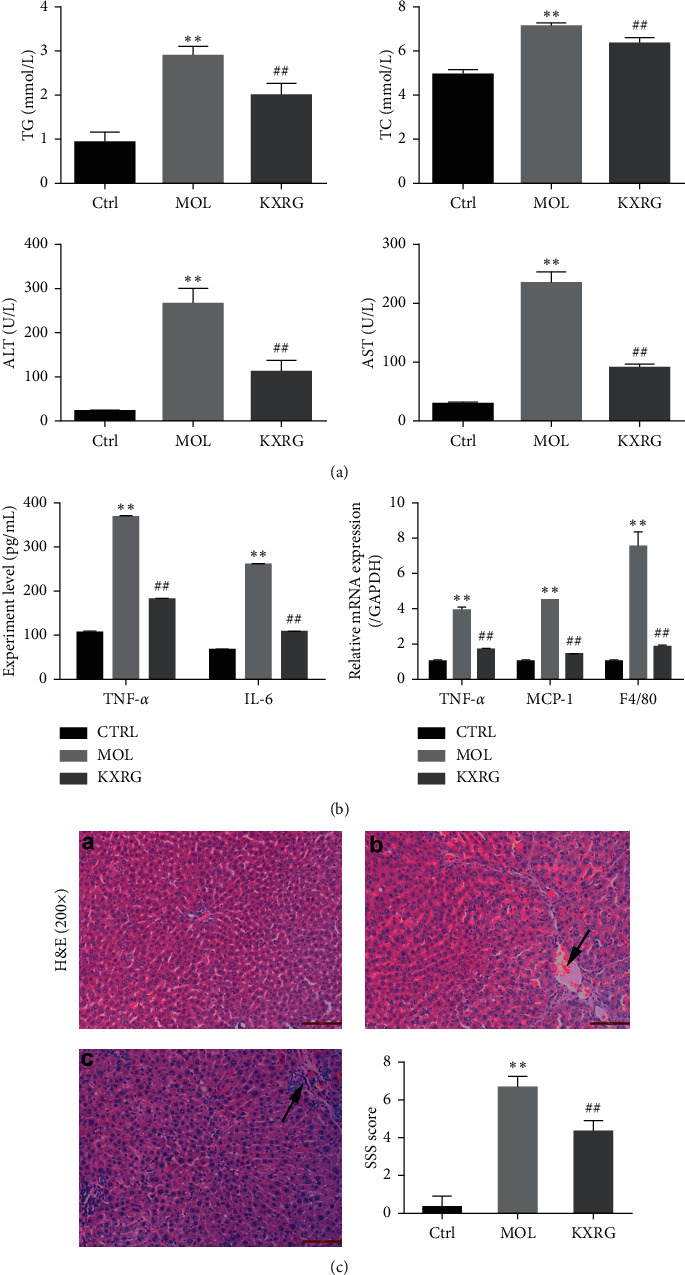
Effect of KXRG on inflammatory cytokines in serum and histopathological changes in liver. CTRL: Control; MOL: Model; KXRG: Kangxian ruangan. (a) Level of TG, TC, ALT and AST, (b) Level of TNF-*α* and IL-6, and mRNA expression of TNF-*α*, MCP-1 and F4/80; (c) Liver histopathological changes and SSS score; (a) Control; (b) Model; (c) KXRG. The results are presented as the mean ± SD (*n* = 8 per group).  ^*∗∗*^*p* < 0.01 compared with CTRL; ^##^*p* < 0.01 compared with MOL. The scale bar is 50 *μ*M (magnification: 200×).

KXRG showed a protective effect on the liver injury, as demonstrated by that KXRG decreased the levels of TG, TC, ALT, and AST increased by MCD (Figure 1(a)) [36]. To evaluate the effect of KXRG on inflammatory factors, we measured the expression levels of TNF-*α* and IL-6 in rats by ELISA and the mRNA expression of TNF-*α*, MCP-1 and F4/80.  Figure 1(b) shows that the levels of TNF-*α*, IL-6 and the mRNA expression of TNF-*α*, MCP-1, and F4/80 in the KXRG treatment group were significantly lower than those in the MOL group (*P* < 0.01). To observe the effect of KXRG on histopathology, the rat liver tissues in each group were stained with H&E. As shown in Figure 1(c), compared with the CTRL group, the hepatic lobule structure in the MOL group was confused and the hepatocytes were disorganized. Severe inflammatory infiltration and hepatocyte necrosis in portal areas can be observed (arrows). Compared with the MOL group, the hepatic lobule structure of the KXRG treatment group was normal and the hepatocytes were neatly arranged. Some hepatocytes have degenerated around the central vein (arrows), the lesion area was smaller, and the degree of liver cell degeneration was lighter. The abnormal proliferation of fibrous tissue was not obvious. In addition, the score of SSS evaluation for liver fibrosis in MOL group was significantly higher than that in CTRL group (Figure 1(c), *P* < 0.01). At the same time, the score in the KXRG group was obviously less than that in the MOL group (*P* < 0.01). These results validate that KXRG significantly prevented the inflammatory response and pathological changes in rats with NAFLD fibrosis induced by the MCD diet.

### 3.2. The Effect of KXRG on Fibrosis in Liver

**Figure 2:**
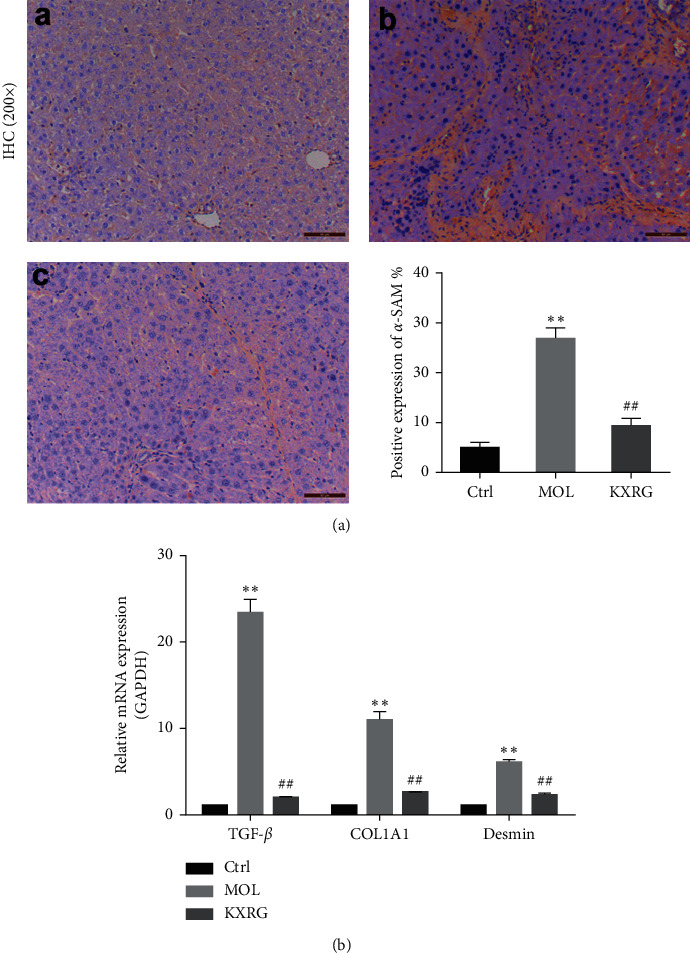
KXRG reduced the expression of *α*-SMA in the liver. CTRL: Control; MOL: Model; KXRG: Kangxian ruangan. (a) Immunohistochemical staining and statistical analysis of the positive expression of *α*-SMA; (b) mRNA expression of TGF-*β*, COL1A1, and Desmin. (a) Control; (b) Model; (c) KXRG.  ^*∗∗*^*p* < 0.01 compared with CTRL; ^##^*p* < 0.01 compared with MOL. The scale bar is 50 *μ*M (magnification: 200×).

To better demonstrate the effect of KXRG on liver fibrosis in rats, the expression of *α*-SMA, an important indicator of fibrosis, in the liver was detected by immunohistochemical staining. As shown in Figure 2(a), the model group exhibited typical tan staining, indicating the high expression level of *α*-SMA. Intuitively, the degree of tan staining in the KXRG treatment group decreased significantly. The quantitative analysis showed that the positive expression of *α*-SMA in the model group increased significantly compared with that in the control group (*P* < 0.01). Compared with the model group, the positive expression of *α*-SMA decreased significantly with KXRG treatment (*P* < 0.01). In addition, the mRNA expression of TGF-*β*, COL1A1, and desmin in rat livers was measured using qRT-PCR. The results indicated that KXRG inhibited the mRNA expression of TGF-*β*, COL1A1, and desmin increased by MCD (Figure 2(b)). These findings suggest that KXRG significantly relieved liver fibrosis by reducing the expression of *α*-SMA, TGF-*β*, COL1A1, and desmin.

### 3.3. The Effect of KXRG on TGF-*β* and TLR4 Signaling Pathways *In Vivo*

**Figure 3:**
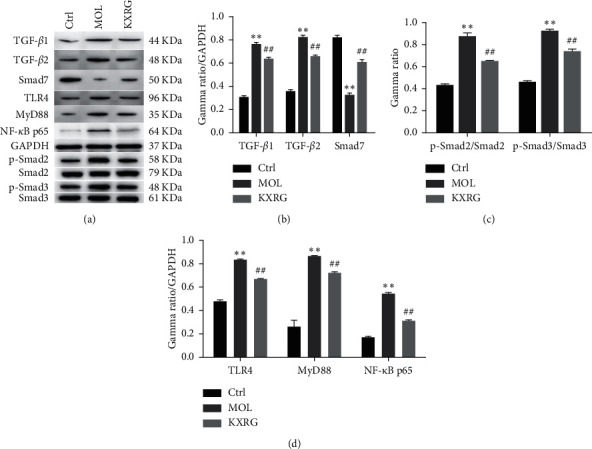
KXRG inhibited the TGF-*β* and TLR4 signaling pathways in rat liver. CTRL: Control; MOL: Model; KXRG: Kangxian ruangan; (a) Western blot of proteins; Statistical analysis of (b) and (c) TGF-*β* signaling-related proteins and (d) TLR4 signaling-related proteins. ^*∗∗*^*p* < 0.01 compared with CTRL; ^##^*p* < 0.01 compared with MOL.

To investigate the correlation between KXRG treatment and TGF-*β* and TLR4 signaling, the protein expression levels of corresponding regulatory factors were detected by western blot (Figure 3(a)**)**. In terms of TGF-*β* signaling, the expression levels of TGF-*β*1, TGF-*β*2, p-Smad2, and p-Smad3 in the KXRG treatment group were significantly lower than those in the MOL group (*P* < 0.01), while Smad7 was significantly increased (*P* < 0.01) (Figure 3(b)/C). On the other hand, in terms of TLR4 signaling, the expression levels of TLR4, MyD88, and NF-*κ*B p65 in the KXRG treatment group were significantly lower than those in the MOL group (*P* < 0.01) (Figure 3(d)). These remarkable results revealed that the KXRG capsules prevented and treated NAFLD fibrosis in rats, possibly via the regulation of the TGF-*β* and TLR4 signaling pathways.

### 3.4. Observation and Identification of HSCs

**Figure 4:**
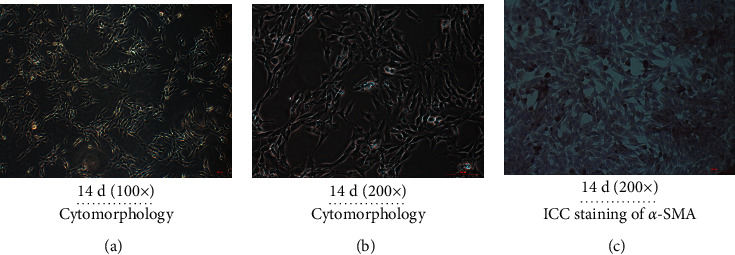
Observation and identification of HSCs. Observation of HSC cytomorphology at 14 d at magnification of (a) 100× and (b) 200×; (c) Identification of HSCs (14 d) by immunohistochemical staining of *α*-SMA.

Cytomorphological identification demonstrated that the separated primary cells did not adhere and showed a microscopic spherical shape, and the lipid droplets were visible in the cytoplasm. After approximately 14 days of culture, the cells transformed into a fibroblastic morphology, mostly irregular polygons, forming clusters with larger cells and extending to the periphery (Figures 4(a) and 4(b)). The results of immunocytochemistry showed that the cytoplasm of *α*-SMA-positive cells in HSCs cultured for 14 days was stained in tan (Figure 4(c)), indicating that the separated and cultured cells were HSCs.

### 3.5. The Effect of KXRG on HSC Proliferation, Apoptosis, and Cycle

**Figure 5:**
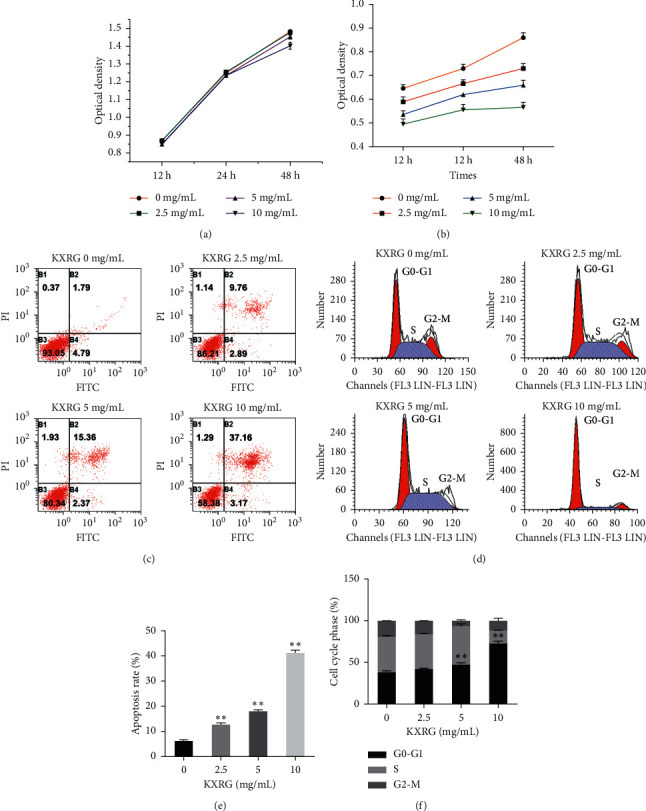
KXRG inhibited HSC proliferation, promoted HSC apoptosis, and led to the arrest of G0-G1 phase in HSCs. KXRG: Kangxian ruangan; (a) Effect of KXRG on LO2 cells proliferation; (b) Effect of KXRG on HSC proliferation; ^*∗*^*p* < 0.05, ^*∗∗*^*p* < 0.01 compared with same treatment group at 12 h; (c) and (d) Effect of KXRG on HSC apoptosis; (e) and (f) Effect of KXRG on HSC cycle; ^*∗∗*^*p* < 0.01 compared with 0 mg/mL KXRG treatment.

The effect of KXRG on LO2 cells and HSC proliferation was assessed by the MTT method. KXRG treatment no more than 24 h showed no effect on proliferation of LO2 cells (Figure 5(a)) and reduced the proliferation ability of HSCs in a concentration-dependent manner, and the inhibitory effect of 10 mg/mL KXRG on HSC proliferation was the most prominent at 24 h (Figure 5(b)). Next, the effect of KXRG on HSC apoptosis was evaluated by Annexin V-FITC/PI staining (Figures 5(c) and 5(d)). Compared with the 0 mg/mL KXRG group, the apoptosis rate of cells in each KXRG treatment group increased significantly (*P* < 0.01), which indicated that KXRG induced cell apoptosis in a dose-dependent fashion at an optimum concentration of 10 mg/mL. Furthermore, the impact of KXRG on the cell cycle was examined by flow cytometry (Figures 5(e) and 5(f)). Compared with the 0 mg/mL KXRG group, incubation with KXRG at 5 mg/mL and 10 mg/mL for 24 h markedly increased the number of cells in the G0-G1 phase (*P* < 0.01). The results demonstrated that 10 mg/mL KXRG significantly inhibited HSC proliferation, promoted HSC apoptosis, and led to the arrest of the G0-G1 phase in HSCs.

### 3.6. The Effect of KXRG on PDGF-Stimulated HSC Proliferation, Collagen Synthesis, and *α*-SMA Expression

**Figure 6:**
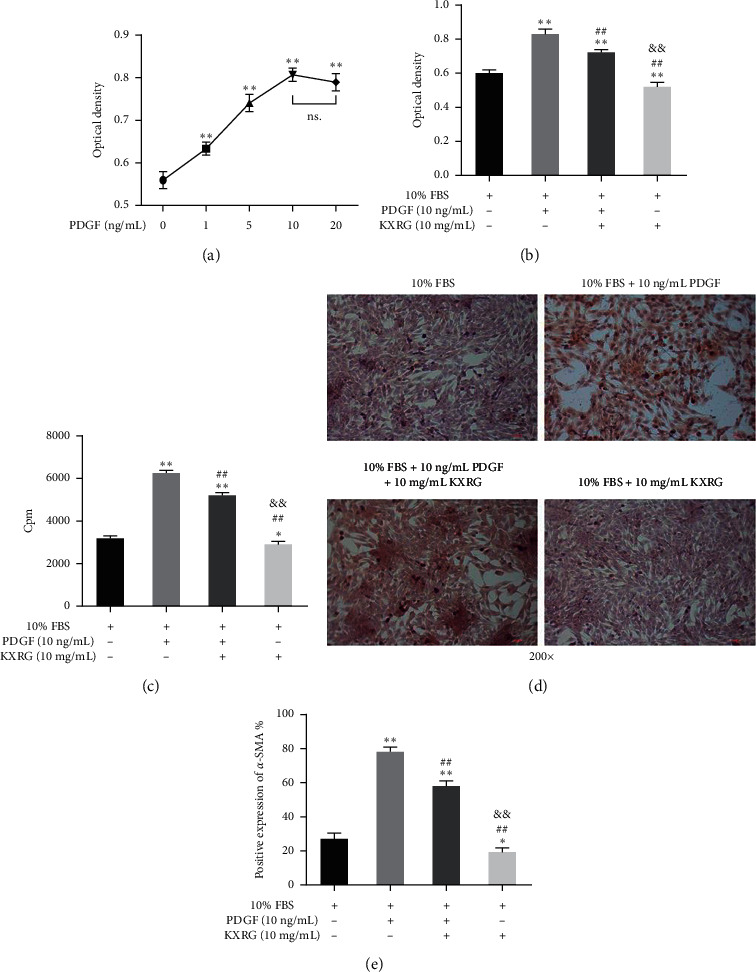
KXRG suppressed proliferation, collagen synthesis, and *α*-SMA expression in HSCs stimulated by PDGF. (a) Effect of PDGF on HSC proliferation; ^*∗∗*^*p* < 0.01 compared with 0 ng/mL PDGF group; (b) Effect of KXRG on HSC proliferation stimulated by PDGF; (c) Effect of KXRG on collagen synthesis in HSCs stimulated by PDGF; (d) and (e) Effect of KXRG on *α*-SMA expression of HSCs stimulated by PDGF; ^*∗∗*^*p* < 0.01 compared with 10% FBS group; ^##^*p* < 0.01 compared with 10% FBS + 10 ng/mL PDGF group; ^&&^*p* < 0.01 compared with 10% FBS + 10 ng/mL PDGF + 10 mg/mL KXRG group.

In this study, PDGF was selected to construct the HSC fibrosis model. Preliminary experimental results showed that 10 ng/mL was the optimum PDGF concentration for the HSC fibrosis model (Figure 6(a)). As shown in Figure 6(b), 10 ng/mL PDGF significantly increased the activity of HSCs (*P* < 0.01), while 10 mg/mL KXRG significantly inhibited the proliferation of PDGF-stimulated HSCs (*P* < 0.01). The effect of KXRG on collagen synthesis was evaluated by ^3^H-proline incorporation assay (Figure 6(c)). At 10 ng/mL, PDGF significantly increased collagen synthesis in HSCs (*P* < 0.01). On the contrary, KXRG played a certain inhibitory effect on collagen synthesis with the stimulation of PDGF. As an important indicator of the degree of fibrosis, *α*-SMA expression was detected by immunocytochemical staining. As shown in Figures 6(d) and 6(e), KXRG significantly reduced the high expression of *α*-SMA in PDGF-induced HSCs (*P* < 0.01). These results indicate that KXRG significantly suppressed proliferation, collagen synthesis, and *α*-SMA expression in PDGF-stimulated HSCs.

### 3.7. The Effect of KXRG on TGF-*β* and TLR4 Signaling Pathways in HSCs

**Figure 7:**
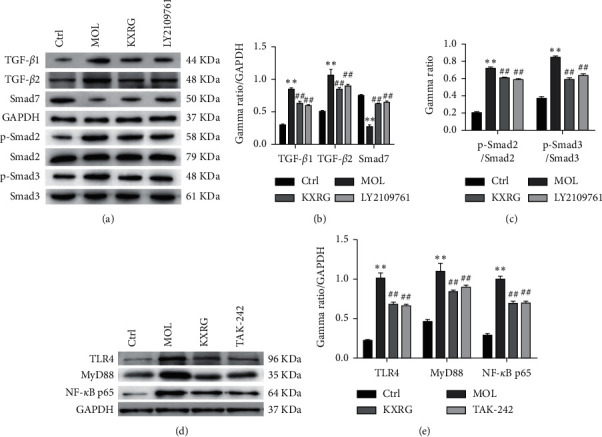
KXRG inhibited TGF-*β* and TLR4 signaling in HSCs. CTRL: Control; MOL: Model; KXRG: Kangxian ruangan; (a) Western blot of TGF-*β* signaling-related proteins; (b) and (c) Statistical analysis of TGF-*β* signaling-related proteins; (d) Western blot of TLR4 signaling-related proteins; (e) Statistical analysis of TLR4 signaling-related proteins; ^*∗∗*^*p* < 0.01 compared with CTRL; ^##^*p* < 0.01 compared with MOL.

To further explore the possible antifibrogenic mechanism of KXRG in hepatic fibrosis, we examined the effects of KXRG on PDGF-stimulated intracellular pathways in HSCs, focusing on TGF-*β* and TLR4 signaling pathways. As indicated in Figures 7(a), 7(b), and 7(c), KXRG significantly reduced the expression of TGF-*β*1, TGF-*β*2, p-Smad2, and p-Smad3 but improved the expression of Smad7 to a certain extent. For the TLR4 signaling pathway, Figures 7(d) and 7(e) demonstrates that KXRG significantly decreased PDGF-induced expression of TLR4, MyD88, and NF-*κ*B p65. In addition, to investigate the correlation between the inhibitory effect of the two pathways and the inhibitory actions of KXRG, HSCs were pretreated with LY2109761, a TGF-*β* receptor inhibitor, and TAK-242, a small-molecule inhibitor of TLR4. Treatment with LY2109761 or TAK-242 resulted in significant inhibition of the expression of related molecules within the corresponding pathway. In particular, it should be noted that KXRG treatment rendered no evident difference compared with inhibitor treatment. These results suggest that part of the action of KXRG may be mediated through the inhibition of the TGF-*β* and TLR4 signaling pathways.

## 4. Discussion

NAFLD is considered to be the most common cause of chronic liver diseases worldwide and is a multifactorial disease with complex pathophysiology, including hepatic steatosis, nonalcoholic steatohepatitis, and subsequent fibrosis, cirrhosis, and even hepatocellular carcinoma [37]. With the improvement in quality of life and the imbalance of diet, NAFLD has gradually become a common disease in developing countries, including China, with high incidence rates [38]. As an essential stage of the development of chronic liver disease into cirrhosis, the reversal of hepatic fibrosis is a top priority in the treatment of NAFLD. Past studies have shown that feeding mice an MCD diet led to the development of steatohepatitis with fibrosis and could serve as a good animal model for NAFLD [39, 40].

Few international research reports on KXRG capsules were found according to a review of past studies. However, from the perspective of both the symptoms and the root of TCM, previous studies have reported the effective hepatoprotective action of KXRG capsules [41]. In addition, as the main form of TCM, TCM formulae have gradually been accepted by various countries for their multitarget treatment [42, 43]. In this study, a rat model of NAFLD fibrosis was successfully established, as demonstrated by the increase of ALT and AST [36]. In addition, liver histology showed hepatic steatosis, necrosis, inflammatory cell infiltration, and a marked increase in hepatocyte apoptosis. In this context, we evaluated whether the involvement of KXRG improves histopathological changes in rat liver induced by MCD feeding.

Proinflammatory cytokines mediate inflammatory reactions and apoptosis. In particular, the cytokine TNF-*α* and MCP-1 are involved in systemic inflammation in acute-phase response and has been shown to play an important role in the progression of steatohepatitis [44, 45]. IL-6 is an inflammatory mediator of liver diseases, including obesity-associated fatty liver [46] and cirrhosis [47]. F4/80 is a specific biomarker of macrophage, which is one of the inflammatory cells [45]. Many recent studies have highlighted the importance of regulatory anti-inflammatory cytokines in controlling the differentiation and function of lymphocytes under steady-state and inflammatory conditions and minimizing tissue damage [48, 49]. In our study, KXRG significantly reduced the expression of TNF-*α* and IL-6 in the fasting serum and mRNA expression of TNF-*α*, MCP-1, and F4/80 in liver tissue of MCD-fed rats. This suggests that the anti-inflammatory effect of KXRG may be related to the inhibition of TNF-*α*, IL-6, MCP-1, and F4/80 expression. Similarly, as important fibrotic markers, *α*-SMA, TGF-*β*, COL1A1, and desmin expression was also inhibited by KXRG treatment, thus inhibiting hepatic fibrosis. These results indicate that KXRG may be a promising agent for the treatment of liver fibrosis.

The activation of resident HSCs has been accepted as a central event in the development of hepatic fibrosis and activated HSCs are considered to be a major target in antifibrotic therapy [7, 50]. These peculiar cells are defined as fat-storing pericytes that constitute 5–8% of all liver cells. Under normal conditions, HSCs are static but are activated by inflammatory stimuli and paracrine interaction with Kupffer cells and become highly proliferative [51]. The ability to synthesize and deposit matrix proteins is closely associated with this proliferative phase. It is generally accepted that this activated phase is denoted by the induction of *α*-SMA in HSCs in experimental models of liver injury [52]. Our studies showed that MCD diet-induced *α*-SMA in liver tissues and PDGF stimulation promoted the expression of *α*-SMA in HSCs. According to many studies, inhibiting activation and proliferation and inducing apoptosis are effective means of clearing activated HSCs, which is important for the elimination of fibrosis [53–55]. In this context, we studied the effect of KXRG on the regulation of HSC proliferation, apoptosis, and cycle *in vitro*. Our data showed that KXRG significantly inhibited HSC proliferation, promoted HSC apoptosis, and led to the arrest of the G0-G1 phase in HSCs. These data suggest that KXRG may block hepatic fibrogenesis via clearance of activated HSCs or a direct inhibitory effect on HSC activation.

Finally, we explored the intracellular pathways that mediate the effects of KXRG on HSC function, focusing on TGF-*β* and TLR4 signaling pathways. In mammals, the TGF-*β* pathway regulates many cellular functions, including cell growth [56], differentiation [56], apoptosis [57], ECM production [58], immunity [59], and embryonic development [60]. The TGF-*β* pathway has therefore become a popular target for drug development. For instance, the expression of TGF-*β*1 was upregulated in experimental animal models of hepatic fibrosis induced by CCl_4_ [61]. Furthermore, the mRNA expression of TGF-*β*1 increased in patients with hepatic fibrosis [62]. With the development and application of drugs that inhibit TGF-*β* signaling, the symptoms of hepatic fibrosis have been ameliorated immensely. Some interesting research has shown that liver fibrogenesis is associated with increased intestinal permeability [63]: lipopolysaccharides (LPS), the most common metabolites of Gram-negative bacteria, signal via TLR pathways. TLRs are innate immune signal receptors that can recognize LPS effectively. Upon TLR activation, cells produce proinflammatory cytokines such as TNF-*α*, IL-6, IL-1, and monocyte chemoattractant protein-1, which further contribute to liver fibrosis [64]. In *in vivo* experiments with TLR4-chimeric mice and LPS challenge, researchers have shown that quiescent HSCs, the main precursors of myofibroblasts in liver tissues, are the predominant targets through which TLR4 ligands promote liver fibrogenesis [65]. On the contrary, mice with deficiencies of components of the TLR4 signaling pathway are less susceptible to liver fibrosis [66]. In addition, TLR4 activation not only upregulates chemokine secretion and induces chemotaxis of Kupﬀer cells but also downregulated the TGF-*β*1 pseudoreceptor Bambi to sensitize HSCs and further allowed unrestricted activation of Kupﬀer cells [67]. A previous study indicated that Oxymatrine alleviated CCl4-induced liver fibrosis by inhibiting TLR4 and TGF-*β*1 signaling pathways [68]. Our results revealed that KXRG, similar to the small-molecule inhibitors LY2109761 and TAK-242, significantly inhibited TGF-*β*1 and TLR4 signaling both *in vitro* and *in vivo*. These results indicate that the antifibrotic property of KXRG may inhibit HSC activity by targeting the TGF-*β*1 and TLR4 signaling pathways.

## 5. Conclusion

Our study demonstrated that KXRG attenuated hepatic fibrosis through multiple mechanisms both *in vitro* and *in vivo*. The inhibition of HSC function by KXRG may be closely correlated with the inhibition of TGF-*β*1 and TLR4 signaling. In addition, KXRG exerted remarkable hepatoprotective activity by suppressing the progression of liver fibrosis. The limitation of our current study is the lack of a positive control group for the treatment effect. These data suggested that KXRG may have the potential as an effective treatment for NAFLD fibrosis and provide novel cellular mechanisms because of its antifibrotic effects.

## Data Availability

The data used to support the findings of this study are included within the article.

## Supplementary Materials

Supplementary Materials Table S1: the Chinese and English version of 12 traditional Chinese medicines in KXRG; Figure S1: UHPLC-QTOF-MS fingerprint of Kangxian ruangan capsule in the positive mode; Table S2: UHPLC-QTOF-MS detection of the chemical composition of Kangxian ruangan capsule in the positive mode; Figure S2: UHPLC-QTOF-MS fingerprint of Kangxian ruangan capsule in the negative mode; Table S3: UHPLC-QTOF-MS detection of the chemical composition of Kangxian ruangan capsule in the negative mode.

## References

[1] Halmos T., Suba I. (2014). [Nonalcoholic fatty liver disease, as a component of the metabolic syndrome, and its causal correlations with other extrahepatic diseases]. *Orvosi Hetilap*.

[2] Perumpail B. J., Khan M. A., Yoo E. R., Cholankeril G., Kim D., Ahmed A. (2014). Clinical epidemiology and disease burden of nonalcoholic fatty liver disease. *World Journal of Gastroenterology*.

[3] Mukherjee P. S., Vishnubhatla S., Amarapurkar D. N. (2015). Etiology and mode of presentation of chronic liver diseases in India: a multi centric study. *PLoS One*.

[4] Lee S. W., Lee T. Y., Yang S. S., Peng Y. C., Yeh H. Z., Chang C. S. (2015). The association of nonalcoholic fatty liver disease and metabolic syndrome in a Chinese population. *Hepatobiliary & Pancreatic Diseases International*.

[5] Younossi Z., Anstee Q. M., Marietti M. (2016). Global burden of NAFLD and NASH: trends, predictions, risk factors and prevention. *Nature Reviews Gastroenterology & Hepatology*.

[6] Maida M., Macaluso F. S., Salomone F., Petta S. (2017). Non-invasive assessment of liver injury in nonalcoholic fatty liver disease: a review of literature. *Current Molecular Medicine*.

[7] Zhang C. Y., Yuan W. G., He P., Lei J. H., Wang C. X. (2018). Liver fibrosis and hepatic stellate cells: etiology, pathological hallmarks and therapeutic targets. *World Journal of Gastroenterology*.

[8] Wree A., Broderick L., Canbay A., Hoffman H. M., Feldstein A. E. (2019). From NAFLD to NASH to cirrhosis-new insights into disease mechanisms. *Nature Reviews Gastroenterology & Hepatology*.

[9] Hardy T., Oakley F., Anstee Q. M., Day C. P. (2014). Nonalcoholic fatty liver disease: pathogenesis and disease spectrum. *Annual Review of Pathology*.

[10] Zoller H., Tilg H. (2019). Nonalcoholic fatty liver disease and hepatocellular carcinoma. *Metabolism*.

[11] Par A., Par G. (2020). [Advances in the pathogenesis of non alcoholic fatty liver disease]. *Orvosi Hetilap*.

[12] Goodman Z. D. (2014). Grading and staging systems for inflammation and fibrosis in chronic liver diseases. *Journal of Hepatology*.

[13] Caussy C., Soni M., Cui J. (2014). Nonalcoholic fatty liver disease with cirrhosis increases familial risk for advanced fibrosis. *Journal of Clinical Investigation*.

[14] Affo S., Yu L. X., Schwabe R. F. (2016). The role of cancer-associated fibroblasts and fibrosis in liver cancer. *Annual Review of Pathology*.

[15] Parkes J., Roderick P., Harris S. (2019). Enhanced liver fibrosis test can predict clinical outcomes in patients with chronic liver disease. *Gut*.

[16] Huang Y., Deng X., Liang J. (2018). Modulation of hepatic stellate cells and reversibility of hepatic fibrosis. *Experimental Cell Research*.

[17] Hong I. H., Park S. J., Goo M. J. (2012). JNK1 and JNK2 regulate alpha-SMA in hepatic stellate cells during CCl4 -induced fibrosis in the rat liver. *Pathology International*.

[18] Zhang L., Liu C., Meng X. M., Huang C., Xu F., Li J. (2013). Smad2 protects against TGF-beta1/Smad3-mediated collagen synthesis in human hepatic stellate cells during hepatic fibrosis. *Molecular and Cellular Biochemistry*.

[19] Wang A., Zhang F., Xu H. (2012). TWEAK/Fn14 promotes proinflammatory cytokine secretion in hepatic stellate cells via NF-kappaB/STAT3 pathways. *Molecular Immunology*.

[20] Lee J. H., Jang E. J., Seo H. L. (2013). Sauchinone attenuates liver fibrosis and hepatic stellate cell activation through TGF-beta/Smad signaling pathway. *Chemico-Biological Interactions*.

[21] Reif S., Lang A., Lindquist J. N. (2015). The role of focal adhesion kinase-phosphatidylinositol 3-kinase-akt signaling in hepatic stellate cell proliferation and type I collagen expression. *Journal of Biological Chemistry*.

[22] Li J., Li X., Xu W. (2013). Antifibrotic effects of luteolin on hepatic stellate cells and liver fibrosis by targeting AKT/mTOR/p70S6K and TGFbeta/Smad signalling pathways. *Liver International*.

[23] Liu P. (2012). Fuzheng huayu capsule in the treatment of liver fibrosis: clinical evidence and mechanism of action. *Chinese Journal of Integrative Medicine*.

[24] Hu N., Guo C., Dai X. (2011). Forsythiae Fructuse water extract attenuates liver fibrosis via TLR4/MyD88/NF-kappaB and TGF-beta/smads signaling pathways. *Journal of Ethnopharmacology*.

[25] Cai F. F., Wu R., Song Y. N. (2011). Yinchenhao decoction alleviates liver fibrosis by regulating bile acid metabolism and TGF-beta/smad/ERK signalling pathway. *Science Reports*.

[26] Liu L., Zhou Y., Dai D., Xia H., Zhao K., Zhang J. (2012). Protective effects of Kangxian ruangan capsule against nonalcoholic fatty liver disease fibrosis in rats induced by MCD diet. *Biomedicine & Pharmacotherapy*.

[27] Luo X., Li H., Ma L. (2011). Expression of STING is increased in liver tissues from patients with NAFLD and promotes macrophage-mediated hepatic inflammation and fibrosis in mice. *Gastroenterology*.

[28] Widjaja A. A., Singh B. K., Adami E. (2008). Inhibiting interleukin 11 signaling reduces hepatocyte death and liver fibrosis, inflammation, and steatosis in mouse models of nonalcoholic steatohepatitis. *Gastroenterology*.

[29] Tanaka N., Takahashi S., Hu X. (2001). Growth arrest and DNA damage-inducible 45alpha protects against nonalcoholic steatohepatitis induced by methionine- and choline-deficient diet. *Biochimica et Biophysica Acta (BBA)-Molecular Basis of Disease*.

[30] Livak K. J., Schmittgen T. D. (2011). Analysis of relative gene expression data using real-time quantitative PCR and the 2 (-Delta Delta C(T)) Method. *Methods*.

[31] Qiu J. F., Zhang Z. Q., Chen W., Wu Z. Y. (2011). Cystamine ameliorates liver fibrosis induced by carbon tetrachloride via inhibition of tissue transglutaminase. *World Journal of Gastroenterology*.

[32] Shen H., Zhang M., Minuk G. Y., Gong Y. (2011). Different effects of rat interferon alpha, beta and gamma on rat hepatic stellate cell proliferation and activation. *BMC Cell Biology*.

[33] Wu F. R., Pan C. X., Rong C. (2014). Inhibition of acid-sensing ion channel 1a in hepatic stellate cells attenuates PDGF-induced activation of HSCs through MAPK pathway. *Molecular and Cellular Biochemistry*.

[34] Melisi D., Ishiyama S., Sclabas G. M. (2008). A novel transforming growth factor beta receptor type I and type II dual inhibitor, as a therapeutic approach to suppressing pancreatic cancer metastasis. *Molecular Cancer Therapeutics*.

[35] Yu X. Y., Qiao S. B., Guan H. S., Liu S. W., Meng X. M. (2015). Effects of visfatin on proliferation and collagen synthesis in rat cardiac fibroblasts. *Hormone and Metabolic Research*.

[36] Zhang B., Lai L., Tan Y. (2014). Hepatoprotective effect of total flavonoids of Mallotus apelta (Lour.) Muell.Arg. leaf against carbon tetrachloride-induced liver fibrosis in rats via modulation of TGF-beta1/Smad and NF-kappaB signaling pathways. *Journal of Ethnopharmacology*.

[37] Bellentani S. (2015). The epidemiology of nonalcoholic fatty liver disease. *Liver International*.

[38] Fan J. G., Farrell G. C. (2011). Epidemiology of nonalcoholic fatty liver disease in China. *Journal of Hepatology*.

[39] Kanuri G., Bergheim I. (2012). In vitro and in vivo models of nonalcoholic fatty liver disease (NAFLD). *International Journal of Molecular Sciences*.

[40] Kim S. B., Kang O. H., Lee Y. S. (2014). Hepatoprotective effect and synergism of bisdemethoycurcumin against MCD diet-induced nonalcoholic fatty liver disease in mice. *PLoS One*.

[41] Zhou Y., Liu L. M., Yu S. M. (2014). Clinical study of Kangxian Ruangan capsule combined with adefovir dipivoxil in treating hepatic fibrosis of chronic hepatistis B patients. *Chinese Journal of Integrated Traditional and Western Medicine on Liver Diseases*.

[42] Del Prete A., Scalera A., Iadevaia M. D. (2012). Herbal products: benefits, limits, and applications in chronic liver disease. *Evidence-Based Complementary and Alternative Medicine*.

[43] Lam P., Cheung F., Tan H. Y., Wang N., Yuen M. F., Feng Y. (2013). Hepatoprotective effects of Chinese medicinal herbs: a focus on anti-inflammatory and anti-oxidative activities. *International Journal of Molecular Sciences*.

[44] Zahran W. E., Salah El-Dien K. A., Kamel P. G., El-Sawaby A. S. (2011). Efficacy of tumor necrosis factor and interleukin-10 analysis in the follow-up of nonalcoholic fatty liver disease progression. *Indian Journal of Clinical Biochemistry*.

[45] Flores-Costa R., Alcaraz-Quiles J., Titos E. (2011). The soluble guanylate cyclase stimulator IW-1973 prevents inflammation and fibrosis in experimental nonalcoholic steatohepatitis. *British Journal of Pharmacology*.

[46] Ma Y., Gao M., Sun H., Liu D. (2011). Interleukin-6 gene transfer reverses body weight gain and fatty liver in obese mice. *Biochimica et Biophysica Acta*.

[47] Reiberger T., Ferlitsch A., Payer B. A. (2014). Non-selective betablocker therapy decreases intestinal permeability and serum levels of LBP and IL-6 in patients with cirrhosis. *Journal of Hepatology*.

[48] Qiu X., Zhang M., Yang X., Hong N., Yu C. (2011). Faecalibacterium prausnitzii upregulates regulatory T cells and anti-inflammatory cytokines in treating TNBS-induced colitis. *Journal of Crohn’s and Colitis*.

[49] du Plessis J., Korf H., van Pelt J. (2013). Proinflammatory cytokines but not endotoxin-related parameters associate with disease severity in patients with NAFLD. *PLoS One*.

[50] Liu Y. W., Chiu Y. T., Fu S. L., Huang Y. T. (2019). Osthole ameliorates hepatic fibrosis and inhibits hepatic stellate cell activation. *Journal of Biomedical Science*.

[51] Li Y., Xiong L., Gong J. (2020). Lyn kinase enhanced hepatic fibrosis by modulating the activation of hepatic stellate cells. *American Journal of Translational Research*.

[52] Jang Y. O., Jun B. G., Baik S. K., Kim M. Y., Kwon S. O. (2018). Inhibition of hepatic stellate cells by bone marrow-derived mesenchymal stem cells in hepatic fibrosis. *Clinical and Molecular Hepatology*.

[53] Li J., Ghazwani M., Liu K. (2019). Regulation of hepatic stellate cell proliferation and activation by glutamine metabolism. *PLoS One*.

[54] Liang L., Yang X., Yu Y. (2015). Babao Dan attenuates hepatic fibrosis by inhibiting hepatic stellate cells activation and proliferation via TLR4 signaling pathway. *Oncotarget*.

[55] Yu H. X., Yao Y., Bu F. T. (2016). Blockade of YAP alleviates hepatic fibrosis through accelerating apoptosis and reversion of activated hepatic stellate cells. *Molecular Immunology*.

[56] Moustakas A., Pardali K., Gaal A., Heldin C. H. (2014). Mechanisms of TGF-beta signaling in regulation of cell growth and differentiation. *Immunology Letters*.

[57] Ding Y., Kim J. K., Kim S. I. (2014). TGF-{beta}1 protects against mesangial cell apoptosis via induction of autophagy. *Journal of Biological Chemistry*.

[58] Schiller M., Javelaud D., Mauviel A. (2016). TGF-beta-induced SMAD signaling and gene regulation: consequences for extracellular matrix remodeling and wound healing. *Journal of Dermatological Science*.

[59] Diener K. R., Need E. F., Buchanan G., Hayball J. D. (2017). TGF-beta signalling and immunity in prostate tumourigenesis. *Expert Opinion on Therapeutic Targets*.

[60] Li G., Khateeb K., Schaeffer E., Zhang B., Khatib H. (2016). Genes of the transforming growth factor-beta signalling pathway are associated with pre-implantation embryonic development in cattle. *Journal of Dairy Research*.

[61] Fan X., Zhang Q., Li S. (2011). Attenuation of CCl4-induced hepatic fibrosis in mice by vaccinating against TGF-beta1. *PLoS One*.

[62] Dropmann A., Dediulia T., Breitkopf-Heinlein K. (2011). TGF-beta1 and TGF-beta2 abundance in liver diseases of mice and men. *Oncotarget*.

[63] Bataller R., Brenner D. A. (2011). Liver fibrosis. *Journal of Clinical Investigation*.

[64] Cong M., Iwaisako K., Jiang C., Kisseleva T. (2012). Cell signals influencing hepatic fibrosis. *International Journal of Hepatology*.

[65] Seki E., De Minicis S., Osterreicher C. H. (2001). TLR4 enhances TGF-beta signaling and hepatic fibrosis. *Nature Medicine*.

[66] Isayama F., Hines I. N., Kremer M. (2014). LPS signaling enhances hepatic fibrogenesis caused by experimental cholestasis in mice. *American Journal of Physiology-Gastrointestinal*.

[67] Aoyama T., Paik Y. H., Seki E. (2010). Toll-like receptor signaling and liver fibrosis. *Gastroenterol Res Pract*.

[68] Zhao H. W., Zhang Z. F., Chai X. (2014). Oxymatrine attenuates CCl4-induced hepatic fibrosis via modulation of TLR4-dependent inflammatory and TGF-beta1 signaling pathways. *International Immunopharmacology*.

