# Supplementary material for: Antifibrotic Effects of Kangxian Ruangan Capsule on Rats with Nonalcoholic Fatty Liver Fibrosis and Hepatic Stellate Cells through Regulation of TGF-β and TLR4 Signaling Pathways
Source: Evid Based Complement Alternat Med. 2021 Aug 10;2021:5649575. doi: 10.1155/2021/5649575 (PMC8371615; doi:10.1155/2021/5649575)
Supplement: Supplementary Materials — Table S1: the Chinese and English version of 12 traditional Chinese medicines in KXRG; Figure S1: UHPLC-QTOF-MS fingerprint of Kangxian ruangan capsule in the positive mode; Table S2: UHPLC-QTOF-MS detection of the chemical composition of Kangxian ruangan capsule in the positive mode; Figure S2: UHPLC-QTOF-MS fingerprint of Kangxian ruangan capsule in the negative mode; Table S3: UHPLC-QTOF-MS detection of the chemical composition of Kangxian ruangan capsule in the negative mode. [file 5649575.f1.docx]

**Anti-fibrotic effects of Kangxian ruangan capsule on** **rats with non-alcoholic fatty liver fibrosis and hepatic stellate cells through regulation of TGF-β and TLR4 signaling pathways**

Liming Liu, Ying Zhou, Dan Dai, Hongmei Xia, Kang Zhao, Jianjun Zhang^*^

Department of Liver Disease, Zhongshan Hospital of Hubei Province

*Corresponding author: Dr. Jianjun Zhang, Department of Liver Disease, Zhongshan Hospital of Hubei Province, No. 26, Zhongshan Avenue, Qiaokou District, Wuhan, Hubei Province, China

Email: sky20182019@yeah.net

Table S1 The Chinese and English version of 12 traditional Chinese medicines in KXRG

| Chinese name | English name | Source |
| --- | --- | --- |
| 茵陈蒿 | *Artemisia capillaris* | Shangdong |
| 丹参 | *Salvia miltiorrhiza* | Shangdong |
| 鳖甲 | *Turtle shell* | Hubei |
| 三七 | *Panax notoginseng* | Yunnan |
| 桃仁 | *Peach seed* | Shangxi |
| 当归 | *Angelica sinensis* | Gansu |
| 莪术 | *Curcuma zedoaria* | Guangxi |
| 炮山甲 | *Parched pangolin scales* | Guangxi |
| 土鳖虫 | *Ground beetle* | Anhui |
| 白术 | *Rhizoma atractylodis macrocephalae* | Anhui |
| 薏苡仁 | *Coix seed* | Guizhou |
| 黄芪 | *Astragalus membranaceus* | Gansu |


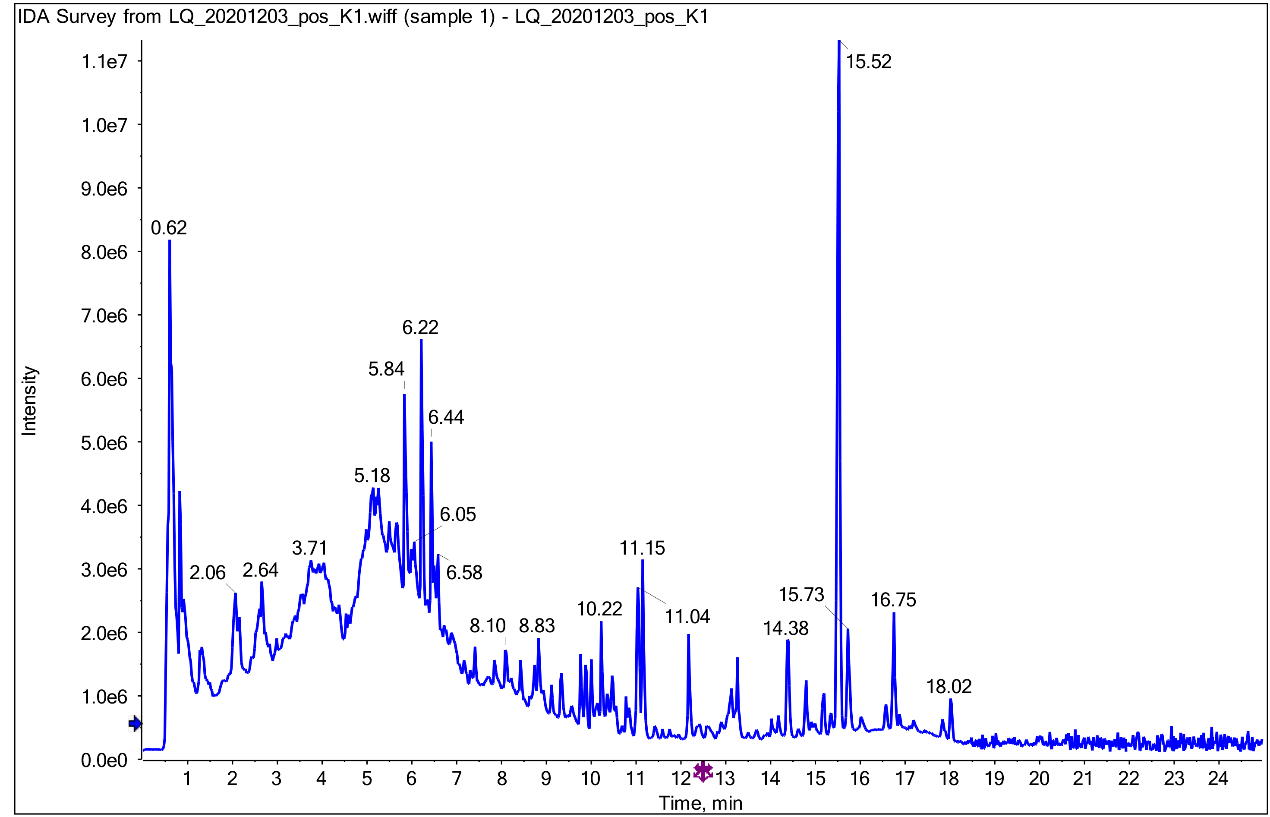


Figure S1. UHPLC-QTOF-MS fingerprint of Kangxian ruangan capsule in the positive mode.

Table S2 UHPLC-QTOF-MS detection of the chemical composition of Kangxian ruangan capsule in the positive mode.

| Compound | Description | m.z | Retention.time..min. |
| --- | --- | --- | --- |
| 0.56_175.1184m/z | DL-Arginine | 175.12 | 0.56 |
| 0.58_689.2092m/z | Stachyose | 689.21 | 0.58 |
| 0.60_365.1048m/z | Sucrose | 365.1 | 0.6 |
| 0.62_162.1123m/z | L-Carnitine | 162.11 | 0.62 |
| 0.64_204.1225m/z | Acetyl-L-carnitine | 204.12 | 0.64 |
| 0.82_130.0855m/z | Pipecolic acid | 130.09 | 0.82 |
| 0.82_182.0809m/z | L-Tyrosine | 182.08 | 0.82 |
| 0.84_284.0998m/z | Isoguanosine | 284.1 | 0.84 |
| 0.87_121.0640m/z | Phenylacetaldehyde | 121.06 | 0.87 |
| 0.96_132.1015m/z | L-Isoleucine | 132.1 | 0.96 |
| 1.00_218.1387m/z | L-Propionylcarnitine | 218.14 | 1 |
| 1.32_120.0807m/z | 2-Amino-1-phenylethanol | 120.08 | 1.32 |
| 1.32_166.0863m/z | L-Phenylalanine | 166.09 | 1.32 |
| 10.22_234.1616n | Valerenic acid | 235.17 | 10.22 |
| 10.29_215.1041m/z | Senkyunolide A | 215.1 | 10.29 |
| 10.35_372.1192n | Tangeritin | 373.13 | 10.35 |
| 10.40_248.1400n | Atractylenolide III | 231.14 | 10.4 |
| 10.75_388.1145n | Artemisetin | 389.12 | 10.75 |
| 10.78_236.1769n | Curcumol | 259.17 | 10.78 |
| 10.97_189.0905m/z | 3-Butylidenephthalide | 189.09 | 10.97 |
| 11.11_278.0938n | Dihydrotanshinone | 301.08 | 11.11 |
| 11.15_190.0989n | Z-Ligustilide | 191.11 | 11.15 |
| 11.59_232.1460n | Isoalantolactone | 233.15 | 11.59 |
| 11.75_294.2192n | 9S-Hydroxy-10E,12Z,15Z-octadecatrienoic acid | 277.22 | 11.75 |
| 12.13_276.0784n | Tanshinone I | 299.07 | 12.13 |
| 12.17_296.1402n | Cryptotanshinone | 319.13 | 12.17 |
| 12.41_231.1375m/z | Dehydrocostus lactone | 231.14 | 12.41 |
| 12.41_311.1249m/z | 10-Angeloylbutylphthalide | 311.12 | 12.41 |
| 12.44_279.2316m/z | 9(10)-Epoxy-12Z-octadecenoic acid | 279.23 | 12.44 |
| 12.57_278.0938n | Dihydroisotanshinone I | 301.08 | 12.57 |
| 12.82_495.3308n | 1-Palmitoyl-sn-glycero-3-phosphocholine | 496.34 | 12.82 |
| 12.99_278.1512n | 1,2-Benzenedicarboxylic acid, bis(2-methylpropyl) ester | 301.14 | 12.99 |
| 13.13_521.3465n | 1-Oleoyl-sn-glycero-3-phosphocholine | 522.35 | 13.13 |
| 13.26_294.1250n | Tanshinone IIA | 317.11 | 13.26 |
| 13.86_323.2817n | Linoleoyl ethanolamide | 346.27 | 13.86 |
| 13.93_352.2601n | Monolinolenin (9c,12c,15c) | 375.25 | 13.93 |
| 14.80_279.2320m/z | Pinolenic acid | 279.23 | 14.8 |
| 14.91_354.2764n | 1-Monolinoleoyl-rac-glycerol | 377.27 | 14.91 |
| 15.19_255.2559n | Palmitamide | 256.26 | 15.19 |
| 15.35_254.2243n | Palmitelaidic acid | 237.22 | 15.35 |
| 15.51_281.2717n | Oleamide | 282.28 | 15.51 |
| 15.77_330.2771n | 2-Palmitoyl-rac-glycerol | 353.27 | 15.77 |
| 16.01_356.2919n | Monoolein | 379.28 | 16.01 |
| 16.57_256.2398n | 2-Hexyldecanoic acid | 257.25 | 16.57 |
| 16.75_282.2556n | cis-Vaccenic acid | 265.25 | 16.75 |
| 17.19_358.3079n | 1-Stearoyl-rac-glycerol | 381.3 | 17.19 |
| 17.83_390.2761n | Bis(2-ethylhexyl) phthalate | 413.27 | 17.83 |
| 18.01_338.3414m/z | Erucamide | 338.34 | 18.01 |
| 2.06_187.0626n | 3-Indoleacrylic acid | 188.07 | 2.06 |
| 2.56_354.0949n | Chlorogenic acid | 377.08 | 2.56 |
| 2.74_395.1306m/z | Eleutheroside B | 395.13 | 2.74 |
| 2.99_144.0806m/z | 1-Naphthalenamine | 144.08 | 2.99 |
| 3.17_151.0388m/z | 2,6-Dihydroxy-4-methylbenzoic acid | 151.04 | 3.17 |
| 3.55_377.1456m/z | (-)-Riboflavin | 377.15 | 3.55 |
| 3.55_594.1574n | Vicenin II | 595.16 | 3.55 |
| 3.62_561.1935m/z | Hyuganoside III | 561.19 | 3.62 |
| 4.69_194.0572n | trans-Ferulic acid | 177.05 | 4.69 |
| 4.96_223.0606m/z | Isofraxidin | 223.06 | 4.96 |
| 5.23_596.1722n | Eriodictyol 7-O-neohesperidoside | 619.16 | 5.23 |
| 5.66_435.1269m/z | Naringenin-7-O-glucoside | 435.13 | 5.66 |
| 5.83_580.1769n | Narirutin | 603.17 | 5.83 |
| 6.10_383.0723m/z | Rosmarinic acid | 383.07 | 6.1 |
| 6.12_609.1797m/z | Neodiosmin | 609.18 | 6.12 |
| 6.14_207.0655m/z | 6,7-Dimethylesculetin | 207.07 | 6.14 |
| 6.21_234.1623n | 13-Hydroxygermacrone | 235.17 | 6.21 |
| 6.21_303.0866m/z | (-)-Homoeriodictyol | 303.09 | 6.21 |
| 6.23_611.1950m/z | Hesperidin | 611.19 | 6.23 |
| 6.43_521.1063m/z | Lithospermic acid | 521.11 | 6.43 |
| 6.43_741.1407m/z | Yunnaneic acid G | 741.14 | 6.43 |
| 6.56_231.1377m/z | Parthenolide | 231.14 | 6.56 |
| 6.81_177.0544m/z | 8-Methoxycoumarin | 177.05 | 6.81 |
| 7.01_237.0756m/z | Dimethylfraxetin | 237.08 | 7.01 |
| 7.39_287.0918m/z | Isosakuranetin | 287.09 | 7.39 |
| 7.75_187.0751m/z | 2,2'-Dihydroxybiphenyl | 187.08 | 7.75 |
| 8.35_292.2031n | 9-Oxo-10E,12Z,15Z-octadecatrienoic acid | 293.21 | 8.35 |
| 8.69_217.0493m/z | 5-Methoxypsoralen | 217.05 | 8.69 |
| 8.69_372.1200n | Isosinensetin | 373.13 | 8.69 |
| 8.85_195.1376m/z | Sedanolide | 195.14 | 8.85 |
| 9.11_260.1044n | 2H-1-Benzopyran-2-one, 7-methoxy, 8-(2-oxoisopentyl) | 261.11 | 9.11 |
| 9.20_373.1267m/z | 5,6,7,3',4'-Pentamethoxyflavone | 373.13 | 9.2 |
| 9.31_342.1095n | 6-Demethoxytangeretin | 343.12 | 9.31 |
| 9.49_395.1456m/z | 6',7'-Dihydroxybergamottin | 395.15 | 9.49 |
| 9.58_217.1581m/z | Polygodial | 217.16 | 9.58 |
| 9.76_402.1308n | Nobiletin | 403.14 | 9.76 |
| 9.83_343.1188m/z | Scutellarein tetramethyl ether | 343.12 | 9.83 |


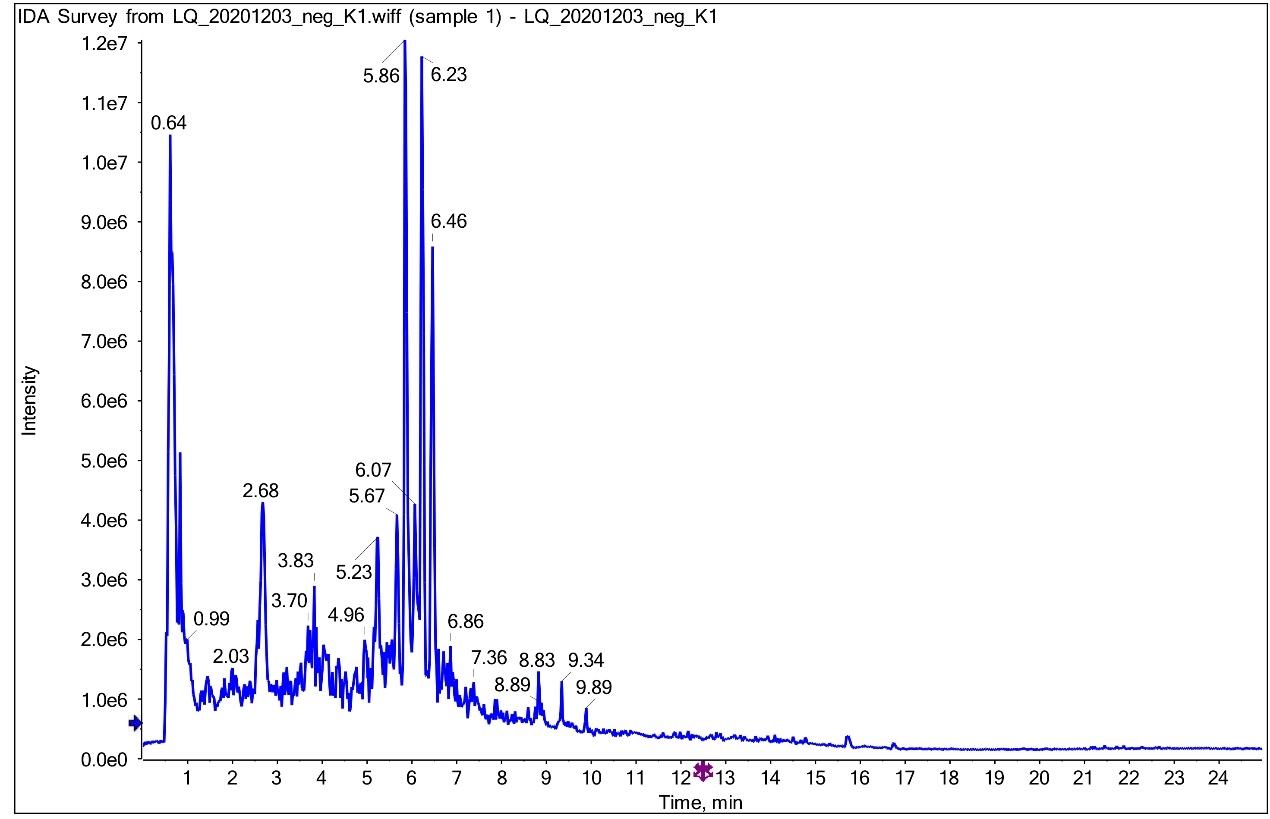


Figure S2. UHPLC-QTOF-MS fingerprint of Kangxian ruangan capsule in the negative mode.

Table S3 UHPLC-QTOF-MS detection of the chemical composition of Kangxian ruangan capsule in the negative mode.

| Compound | Description | m.z | Retention time (min) |
| --- | --- | --- | --- |
| 1.49_198.0528n | Danshensu | 197.05 | 1.49 |
| 3.41_402.1513n | Icariside F2 | 447.15 | 3.41 |
| 0.83_192.0265n | Citric acid | 191.02 | 0.83 |
| 6.22_610.1889n | Hesperidin | 609.18 | 6.22 |
| 4.43_166.0626n | DL-3-Phenyllactic acid | 165.06 | 4.43 |
| 5.84_580.1775n | Narirutin | 579.17 | 5.84 |
| 0.61_342.1148n | Sucrose | 387.11 | 0.61 |
| 6.09_267.0653m/z | 7-Hydroxy-3-(4-hydroxyphenyl)-4-methylcoumarin | 267.07 | 6.09 |
| 6.00_515.1190m/z | Cynarin | 515.12 | 6 |
| 6.04_187.0975m/z | Azelaic acid | 187.1 | 6.04 |
| 6.84_493.1125m/z | Salvianolic acid A | 493.11 | 6.84 |
| 6.46_717.1462m/z | Salvianolic acid B | 717.15 | 6.46 |
| 5.01_204.0658m/z | DL-Indole-3-lactic acid | 204.07 | 5.01 |
| 5.07_463.0875m/z | Hyperoside | 463.09 | 5.07 |
| 5.67_515.1195m/z | Isochlorogenic acid b | 515.12 | 5.67 |
| 5.80_477.1024m/z | Nepetin 7-glucoside | 477.1 | 5.8 |
| 5.76_507.1117m/z | Syringetin 3-galactoside | 507.11 | 5.76 |
| 5.67_447.0935m/z | Astragalin | 447.09 | 5.67 |
| 5.62_623.1605m/z | Narcissin | 623.16 | 5.62 |
| 5.53_537.1032m/z | Isosalvianolic acid C | 537.1 | 5.53 |
| 5.49_593.1503m/z | Kaempferol 3-O-rutinoside | 593.15 | 5.49 |
| 5.62_417.0810m/z | Salvianolic acid D | 417.08 | 5.62 |
| 12.43_295.2273m/z | 12(13)-Epoxy-9Z-octadecenoic acid | 295.23 | 12.43 |
| 10.23_293.1750m/z | [6]-Gingerol | 293.17 | 10.23 |
| 12.16_265.1474m/z | Dodecyl sulfate | 265.15 | 12.16 |
| 11.17_313.2374m/z | 9,10-Dihydroxy-12Z-octadecenoic acid | 313.24 | 11.17 |
| 7.22_491.0979m/z | Salvianolic acid C | 491.1 | 7.22 |
| 7.06_201.1132m/z | Sebacic acid | 201.11 | 7.06 |
| 6.97_285.0399m/z | Luteolin | 285.04 | 6.97 |
| 6.95_263.1288m/z | 2-cis-4-trans-Abscisic acid | 263.13 | 6.95 |
| 8.19_315.0506m/z | Quercetin 3'-methyl ether | 315.05 | 8.19 |
| 7.79_271.0606m/z | Naringenin | 271.06 | 7.79 |
| 7.88_227.1286m/z | trans-Traumatic acid | 227.13 | 7.88 |
| 2.09_339.0693m/z | Esculin | 339.07 | 2.09 |
| 2.07_203.0824m/z | L-Tryptophan | 203.08 | 2.07 |
| 2.25_625.1411m/z | Herbacetin-3,8-diglucopyranoside | 625.14 | 2.25 |
| 2.07_181.0505m/z | p-Hydroxyphenyllactic acid | 181.05 | 2.07 |
| 2.32_787.1928m/z | Quercetin-3-O-β-D-glucose-7-O-β-D-gentiobioside | 787.19 | 2.32 |
| 1.75_201.1243m/z | L-Alanyl-L-norleucine | 201.12 | 1.75 |
| 1.75_187.1088m/z | Glycyl-L-norleucine | 187.11 | 1.75 |
| 1.86_353.0867m/z | Neochlorogenic acid | 353.09 | 1.86 |
| 1.68_153.0191m/z | 3,4-Dihydroxybenzoic acid | 153.02 | 1.68 |
| 2.34_137.0238m/z | 3,4-Dihydroxybenzaldehyde | 137.02 | 2.34 |
| 2.87_177.0192m/z | Esculetin | 177.02 | 2.87 |
| 3.03_159.0661m/z | Pimelic acid | 159.07 | 3.03 |
| 2.99_179.0346m/z | Caffeic acid | 179.03 | 2.99 |
| 2.63_175.0611m/z | 2-Isopropylmalic acid | 175.06 | 2.63 |
| 0.70_549.1639m/z | D-(+)-Raffinose | 549.16 | 0.7 |
| 0.90_147.0299m/z | D-(-)-Citramalic acid | 147.03 | 0.9 |
| 0.61_165.0410m/z | D-Arabinonic acid | 165.04 | 0.61 |
| 0.61_191.0552m/z | (-)-Quinic acid | 191.06 | 0.61 |
| 0.61_195.0506m/z | D-Gluconic acid | 195.05 | 0.61 |
| 0.63_179.0558m/z | D-Fructose | 179.06 | 0.63 |
| 0.63_167.0209m/z | Uric acid | 167.02 | 0.63 |
| 0.63_133.0138m/z | DL-Malic acid | 133.01 | 0.63 |
| 0.65_191.0194m/z | Mucic acid | 191.02 | 0.65 |
| 1.42_167.0348m/z | Homogentisic acid | 167.03 | 1.42 |
| 1.52_218.1028m/z | Pantothenic acid | 218.1 | 1.52 |
| 1.29_164.0713m/z | L-Phenylalanine | 164.07 | 1.29 |
| 4.52_463.0875m/z | Quercetin 3-glucoside | 463.09 | 4.52 |
| 4.23_579.1699m/z | Naringin | 579.17 | 4.23 |
| 4.86_609.1447m/z | Rutin | 609.14 | 4.86 |
| 4.97_595.1658m/z | Eriocitrin | 595.17 | 4.97 |
| 4.68_193.0502m/z | trans-Ferulic acid | 193.05 | 4.68 |
| 4.57_191.0345m/z | 6-Hydroxy-7-methoxycoumarin | 191.03 | 4.57 |
| 4.57_173.0819m/z | Suberic acid | 173.08 | 4.57 |
| 4.77_206.0822m/z | N-Acetyl-L-phenylalanine | 206.08 | 4.77 |
| 3.26_121.0293m/z | 4-Hydroxybenzaldehyde | 121.03 | 3.26 |
| 3.17_167.0347m/z | 3,5-Dihydroxy-4-methylbenzoic acid | 167.03 | 3.17 |
| 3.55_593.1496m/z | Safflor yellow A | 593.15 | 3.55 |
| 3.94_367.1026m/z | 3-O-Feruloylquinic acid | 367.1 | 3.94 |
| 4.03_163.0396m/z | trans-2-Hydroxycinnamic acid | 163.04 | 4.03 |
| 3.73_131.0708m/z | (+)-2-Hydroxyisocaproic acid | 131.07 | 3.73 |
